# Diaphragm echodensity in mechanically ventilated patients: a description of technique and outcomes

**DOI:** 10.1186/s13054-021-03494-9

**Published:** 2021-02-16

**Authors:** Benjamin Coiffard, Stephen Riegler, Michael C. Sklar, Martin Dres, Stefannie Vorona, W. Darlene Reid, Laurent J. Brochard, Niall D. Ferguson, Ewan C. Goligher

**Affiliations:** 1 Inter-Departmental Division of Critical Care Medicine, University of Toronto, Toronto, Canada; 2 Division of Respirology, Department of Medicine, University Health Network, Toronto, Canada; 3 Keenan Centre for Biomedical Research, Li Ka Shing Knowledge Institute, St. Michael’s Hospital, Toronto, Canada; 4 AP-HP, Service de Pneumologie, Médecine Intensive–Réanimation (Département “R3S”), Groupe Hospitalier Pitié-Salpêtrière Charles Foix, Paris, France; 5 Department of Physical Therapy, University of Toronto, Toronto, Canada; 6 Toronto General Hospital Research Institute, Toronto, Canada

**Keywords:** Ultrasonography, Acute respiratory failure, Artificial respiration, Ventilator weaning

## Abstract

**Background:**

Acute increases in muscle sonographic echodensity reflect muscle injury. Diaphragm echodensity has not been measured in mechanically ventilated patients. We undertook to develop a technique to characterize changes in diaphragm echodensity during mechanical ventilation and to assess whether these changes are correlated with prolonged mechanical ventilation.

**Methods:**

Diaphragm ultrasound images were prospectively collected in mechanically ventilated patients and in 10 young healthy subjects. Echodensity was quantified based on the right-skewed distribution of grayscale values (50th percentile, ED50; 85^th^ percentile, ED85). Intra- and inter-analyzer measurement reproducibility was determined. Outcomes recorded included duration of ventilation and ICU complications (including reintubation, tracheostomy, prolonged ventilation, or death).

**Results:**

Echodensity measurements were obtained serially in 34 patients comprising a total of 104 images. Baseline (admission) diaphragm ED85 was increased in mechanically ventilated patients compared to younger healthy subjects (median 56, interquartile range (IQR) 42–84, vs. 39, IQR 36–52, *p* = 0.04). Patients with an initial increase in median echodensity over time (≥ + 10 in ED50 from baseline) had fewer ventilator-free days to day 60 (*n* = 13, median 46, IQR 0–52) compared to patients without this increase (*n* = 21, median 53 days, IQR 49–56, unadjusted *p* = 0.03). Both decreases and increases in diaphragm thickness during mechanical ventilation were associated with increases in ED50 over time (adjusted *p* = 0.03, conditional *R*^2^ = 0.80) and the association between increase in ED50 and outcomes persisted after adjusting for changes in diaphragm thickness.

**Conclusions:**

Many patients exhibit increased diaphragm echodensity at the outset of mechanical ventilation. Increases in diaphragm echodensity during the early course of mechanical ventilation are associated with prolonged mechanical ventilation. Both decreases and increases in diaphragm thickness during mechanical ventilation are associated with increased echodensity.

## Background

Point-of-care ultrasound is used to evaluate skeletal muscle structure and function in critically ill patients [1, 2]. Sonography allows assessment of muscle quantity (thickness), muscle contractility (contractile thickening and shortening) and muscle quality (echodensity). Changes in muscle echodensity (also referred to as echogenicity) can be assessed using grayscale analysis to quantify changes in muscle echotexture [3, 4]. Muscle echodensity (the sonographic property of signal reflection) is normally low. Healthy muscle tissue usually appears dark, almost black, because it contains little fibrous tissue with minimal sound reflection. In disease, replacement of muscle with fat or fibrous tissue increases echodensity and muscle appears ‘brighter’ [5–7]. Moreover, increases in echodensity correlate with muscle fiber degeneration and necrosis in experimental animal models of muscle injury [8, 9]. Increases in echodensity develop with acute muscle injury in athletes, with progression of chronic muscular disease states such as muscular dystrophy, and with muscular inflammation, necrosis, and weakness in critically ill patients [8, 10–12].

Diaphragm structure and function are known to deteriorate during mechanical ventilation. Both acute decreases and increases in diaphragm thickness during mechanical ventilation have been documented repeatedly using point-of-care ultrasound [13–15]; these sonographic findings are associated with poor clinical outcomes [16]. It is unknown whether diaphragm echodensity changes during mechanical ventilation and whether this provides additional independent information about clinically important changes in muscle structure and function during mechanical ventilation and critical illness. Similar assessments have been performed for analyzing the quadriceps echodensity in intensive care unit (ICU) patients and we adapted these measurements to assess diaphragm echodensity [8].

In a previously published cohort study of diaphragm ultrasound [16], we undertook to develop a technique for assessing diaphragm echodensity in ventilated patients, to characterize the evolution of diaphragm echodensity over time during the early course of mechanical ventilation, and to assess its relation to clinical outcome.

## Methods

### Study population and setting

This study was a secondary analysis of a previously published cohort study in mechanically ventilated patients at Toronto General Hospital and St. Michael’s Hospital, located in Toronto, Canada. The study was designed to assess changes in diaphragm thickness over time; during the latter portion of the study we proposed to assess changes in diaphragm echodensity during mechanical ventilation and began collecting B mode images for echodensity analysis.

Patients were enrolled within 36 h of intubation but were ineligible if liberation from mechanical ventilation was expected within 24 h, or if they had received more than 48 h of mechanical ventilation in the past 6 months. The cohort is described in detail elsewhere [16].

Patients from this cohort were included in the present analysis if the following criteria were met: (a) stored B-mode images were available for analysis; (b) the entirety of the pleural and peritoneal membranes were clearly demarcated across the entire image, allowing the diaphragm to be distinguished from surrounding tissues with confidence; (c) images of acceptable quality (criteria a and b) were available for more than one study day; (d) use of consistent gain and frequency settings across the images obtained from the same patient over time. All diaphragm images up to day 5 were analysed.

In addition, 10 healthy subjects (non-smoking, no history of cardio-pulmonary disease) were enrolled to form a control group.

### Measurement of diaphragm thickness and echodensity

The method to collect diaphragm ultrasound images, to measure diaphragm thickness, and to calculate diaphragm thickening fraction was described in the original paper [16, 17]. B-mode images were collected as close to end-expiration as possible. To standardize ultrasound gain and frequency for echodensity measurements, B-mode images were obtained after restoring the ultrasound device settings to start-up, pre-set default values. Over time, three different ultrasound machines were used (Phillips Sparq, Mindray, Fujifilm Sonosite), but the same machine was used for all images collected from each individual patient.

Diaphragm echodensity was quantified by performing a grayscale histogram analysis in ImageJ (National Institutes of Health, Bethesda, MD, USA) (Additional file 1: A). Grayscale histogram analysis to quantify echodensity has been described elsewhere [18–20]; values range between 0 (black) and 255 (white). The analysis was performed using the trace method [20] by selecting the largest free-form area devoid of artifacts between (but excluding) the pleural and peritoneal membranes (Additional file 1: B). A grayscale frequency histogram was generated for the selected region. As depicted in Additional file 1: C, the distribution of echodensity for the selected region is right-skewed and was quantified using two different parameters: the 50_th_ percentile (ED50), and the 85_th_ percentile (ED85). The 50_th_ and 85_th_ percentile thresholds were chosen as they were deemed to represent the center and upper tail of the grayscale distribution, respectively, for each image. We also defined the percentage of pixels above a grayscale value of 65 (high echodensity area, HEA65). The value of 65 (upper limit of normal for echodensity) was determined based on the 95_th_ percentile grayscale value of the average grayscale histogram obtained from the ten healthy subjects (see “Results”).

### Technical assessment of measurement reliability

To assess the repeatability and reproducibility of echodensity measurements, the principal analyzer trained a secondary analyzer using 30 randomly selected images. Both analyzers then analyzed an additional 30 randomly selected images. Intra-analyzer repeatability and inter-analyzer reproducibility of ED50 were quantified by the method of Bland and Altman [21].

To assess whether echodensity measurements were affected by the timing of the respiratory cycle (which may be difficult to standardize in B-mode), echodensity measurements were obtained on single frames representing end-expiration and end-inspiration in 15 randomly selected DICOM files capturing an entire respiratory cycle.

### Patient characteristics and outcomes

Demographic data, comorbidities, admission diagnosis, baseline severity of illness (Simplified Acute Physiology Score [SAPS] II) [22], ventilator settings, arterial blood gas tensions, criteria for sepsis [23], Riker Sedation-Agitation Scale (SAS) [24], exposure to neuromuscular blockade, and Sequential Organ Failure Assessment (SOFA) [25] scores were extracted from the study database. The following outcomes were also extracted: extubation, reintubation, tracheostomy, ICU discharge, hospital discharge, and death. Liberation from ventilation was defined as separation from the ventilator (extubation or tracheostomy mask breathing for 24 h) without resumption of invasive ventilatory support during the ICU admission.

The duration of ventilation was the time from intubation until liberation from ventilation (or death). Ventilator-free days were computed to 60 days; patients who required more than 60 days of ventilatory support or who died on or before day 60 were assigned 0 days. Complications of acute respiratory failure were defined as the occurrence of any of the following events: reintubation, tracheostomy, prolonged ventilation (> 14 days), or death [16].

Investigators responsible for analysis of diaphragm ultrasound images were blinded to patient outcomes. Clinicians responsible for medical decisions including weaning were not aware of ultrasound measurement data. Routine weaning practices were similar across participating ICUs but were not uniformly standardized for the study [16].

### Statistical analysis

Continuous variables were expressed as mean values (± standard deviation) or median values with interquartile ranges (IQR), according to the distribution (Shapiro–Wilk test). Discrete variables are expressed as percentage values.

Associations between clinical characteristics or outcomes and baseline median echodensity at baseline were assessed using linear or logistic regression models. Owing to the relatively lower number of patients in whom echodensity measurements were available, no multivariable adjustments were performed.

Patients in whom ED50 increased by at least 10 points in grayscale value from baseline at any time over the first 5 days of ventilation were classified as having an increase in echodensity. Patients who did not develop a 10-point increase at any time over the first five days were classified as unchanged. This threshold for categorization (10-point increase in ED50) was selected based on the inter-analyzer limit of agreement for ED50 measurement in this study (see “Results”). To mitigate against time-dependent confounding, patients were classified on the first day that the change in ED50 exceeded + 10 from baseline ED50. Comparisons between the two groups were performed using the Mann–Whitney test for continuous variables and using a chi-squared test for categorical variables. The relationship between the development of an increase in echodensity during mechanical ventilation and outcomes was assessed by linear and logistic regression. To address potential confounding, the association between echodensity and prolonged mechanical ventilation was adjusted for the initial change in diaphragm thickness from baseline. The relationship between decreases or increases in diaphragm thickness with ED50 change over time was fit using restricted cubic splines (3 knots) in linear regression, adjusted for daily fluid balance.

To address potential immortal time bias we performed a sensitivity analysis restricted to patients who remained ventilated at least 3 days (to only analyze diaphragm images while under mechanical ventilation which is the main suspected risk factor for diaphragm injury).

All statistical analyses were conducted using R version 3.5.1 (www.R-project.org).

## Results

### Population

Of 191 mechanically ventilated patients in the cohort, echodensity measurements were attempted for the last 41 patients. Of these, images were available for 34 patients comprising a total of 104 diaphragm images over the study period (4 patients had only one day of imaging, 1 patient was excluded due to inconsistent gain and frequency settings, 2 patients were excluded due to low-quality images). Patient characteristics are reported in Table 1. The healthy control group was comprised of 5 women and 5 men with a median age of 27 years, IQR 25–34 years.

**Table 1:** Baseline and change echodensity over time in relation to patient characteristics

Characteristics	Population	Relation with baseline ED50			Relation with change in ED50 from baseline		
	*n* = 34	Coefficient	CI 95%	*p*	≤ 10-point change in ED50, * n* = 21 (62%)	> 10-point increase in ED50, * n* = 13 (38%)	*p*
Age, yr, mean (SD)	59 (16)	0.13	(− 0.46; 0.71)	0.68	59 (16)	59 (17)	0.94
Female sex, *n* (%)	10 (30)	9	(− 29; 11)	0.39	5 (24)	5 (38)	0.60
Co-morbidities, *n* (%)							
COPD	8 (24)	− 15	(− 37; 7)	0.20	4 (19)	4 (31)	0.62
Asthma	**3 (9)**	**35**	(4; 67)	0.04	1 (5)	2 (15)	0.62
ILD	10 (30)	− 20	(− 40; 0)	0.05	6 (29)	4 (31)	1.00
OSA	3 (9)	− 8	(− 42; 26)	0.64	3 (14)	0 (0)	0.46
CHF	2 (6)	− 15	(− 56; 21)	0.47	1 (5)	1 (8)	1.00
Cirrhosis	**5 (15)**	**34**	(10; 58)	0.01	3 (14)	2 (15)	1.00
CKD	6 (18)	5	(− 20; 29)	0.71	3 (14)	3 (23)	0.85
Immunocompromise	10 (30)	16	(− 4; 37)	0.13	5 (24)	5 (38)	0.50
Diabetes	10 (30)	4	(− 17; 25)	0.71	5 (24)	5 (38)	0.50
SOFA, mean over first 72 h, mean (SD)	11 (3)	0.49	(− 2.4; 3.4)	0.74	11 (3)	11 (3)	0.98
SAPS II, mean (SD)	52 (15)	− 0.09	(− 0.73; 0.55)	0.78	52 (16)	51 (13)	0.80
Primary cause of acute respiratory failure, *n* (%)							
Cardiovascular	2 (6)	Reference	1 (5)	1 (8)	0.52		
Respiratory	7 (21)	15	(− 28; 58)	0.33	4 (19)	3 (23)	
Sepsis (nonpulmonary)	5 (15)	12	(− 33; 56)		3 (14)	2 (15)	
Neurologic	2 (6)	45	(− 8; 99)		1 (5)	1 (8)	
Postoperative	6 (18)	− 5	(− 48; 39)		6 (29)	0 (0)	
Post-transplantation	9 (26)	− 1	(− 42; 41)		5 (24)	4 (31)	
Other (hepatic, renal)	3 (9)	− 5	(− 54; 43)		1 (5)	2 (15)	
Use of NIV prior to intubation	4 (12)	3	(− 26; 32)	0.82	2 (10)	2 (15)	0.96
Primary cause of acute respiratory failure, *n* (%)							
Non-respiratory	15 (44)	Reference	8 (38)	7 (54)	0.59		
Respiratory	19 (56)	− 9	(− 28; 9)	0.34	13 (62)	6 (46)	
Sepsis-3 criteria present in first 48 h, *n* (%)	31 (91)	16	(− 17; 49)	0.35	19 (90)	12 (92)	1.00
Comorbidity at baseline (≥ 1), *n* (%)	26 (76)	8	(− 14; 30)	0.47	14 (67)	12 (92)	0.19
Baseline PaO2/FiO2, mmHg	159 (116–241)	0.06	(− 0.02; 0.15)	0.17	138 (105–212)	168 (124–253)	0.29
Cumulative fluid balance on Day 1 of ventilation, L	0.8 (− 0.3–4.5)	− 0.2	(− 1.4; 1.0)	0.72	2.1 (0.1–5.1)	0.3 (− 0.7–0.9)	0.36
Cumulative fluid balance on Day 3 of ventilation, L, mean (SD)	4.1 (5.2)	1.2	(− 1; 3)	0.24	3.7 (6.2)	4.6 (3.5)	0.60
Initial diaphragm thickness, mm	2.3 (2.1–2.5)	16	(− 3; 35)	0.10	2.3 (2.1–2.6)	2.2 (1.9–2.4)	0.49
Diaphragm thickening fraction (%), mean over first 72 h	9 (6–15)	− 0.4	(− 1.5; 0.8)	0.56	9 (6–15)	10 (9–16)	0.31
Baseline ventilator settings							
Mode of ventilaton, *n* (%)							
Controlled	32 (94)	Reference	20 (95)	12 (92)	1.0		
Partially assisted	2 (6)	− 27	(− 66; 12)	0.18	1 (5)	1 (8)	
Ventilator settings							
Vt, ml/kg PBW	6.8 (6.2–7.2)	− 0.7	(− 1.6; 0.2)	0.15	7.0 (6.4–7.4)	6.7 (5.7–7.0)	0.18
Peak airway pressure, cmH2O, mean (SD)	23 (5)	− 0.6	(− 2.0; 0.9)	0.45	23 (5)	24 (4)	0.52
Set inspiratory pressure above PEEP, cmH2O*	14 (13–16)	1.2	(− 1.5; 3.9)	0.39	14 (13–16)	15 (14–18)	0.41
PEEP, cmH2O	8 (6–10)	− 0.4	(− 3.2; 2.3)	0.75	8 (5–10)	8 (8–10)	0.84
Frequency, min-1, mean (SD)	20 (5)	0.7	(− 1.0; 2.3)	0.43	20 (4)	20 (6)	0.80
FiO2	0.5 (0.4–0.6)	− 0.2	(− 3.0; 2.6)	0.90	0.5 (0.4–0.6)	0.5 (0.4–0.8)	0.93
Arterial blood gas tensions							
pH	7.36 (7.31–7.39)	− 0.09	(− 1.4; 1.2)	0.90	7.36 (7.31–7.41)	7.37 (7.32–7.39)	0.84
PaCO2, mmHg, mean (SD)	42 (7)	− 0.4	(− 1.7; 0.8)	0.50	42 (7)	41 (7)	0.54
PaO2, mmHg, mean (SD)	104 (25)	− 0.6	(− 1.6; 0.3)	0.19	98 (25)	111 (24)	0.14

The respiratory primary cause of acute respiratory failure comprises lung transplantation, postoperative pulmonary endarterectomy, chronic lung rejection, exacerbation of interstitial lung disease, pneumonia, and aspiration; non-respiratory are the other diagnoses

CHF = chronic heart failure; CKD = chronic kidney disease; COPD = chronic obstructive pulmonary disease; ILD = interstitial lung disease; NIV = non-invasive ventilation; OSA = obstructive sleep apnea; PBW = predicted body weight; PEEP = positive end-expiratory pressure; SAPS = Simplified Acute Physiology Score; SAS = Riker Sedation-Agitation Scale; SOFA = Sequential Organ Failure Assessment

All numerical distributions are reported as median (interquartile range) unless otherwise noted

^*^Set inspiratory pressure above PEEP = peak airway pressure—PEEP

### Intra- and inter-analyzer reproducibility of echodensity measurements

ED50 was mean 44 (standard deviation 27) across all mechanically ventilated patients at ICU admission. The average difference in ED50 between analyzers was -1.5 (limits of agreement -9 to 6) (Additional file 2: panel A). The average difference in echodensity between images within the same analyzer was -3 (limits of agreement -16–10) (Additional file 2: panel B). Within a single respiratory cycle, ED50 measured by the same analyzer differed between end-expiration and peak inspiration by mean -1 (standard deviation 4) (Additional file 2: panel C).

Reproducibility according to the ultrasound machines (Philips, Mindray, Sonosite) is described in Additional file 3.

### Diaphragm echodensity in healthy subjects and ventilated patients at baseline

**Fig. 1:**
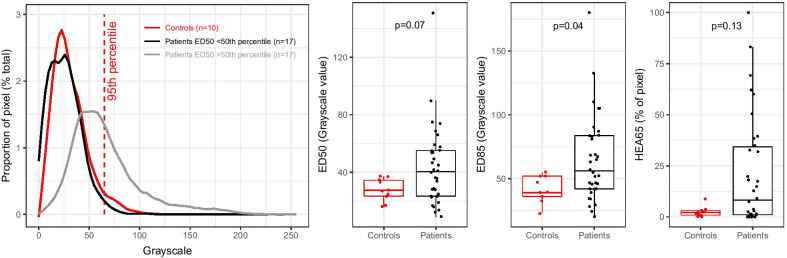
Echodensity histograms of controls and patients at ICU admission. The histograms (left graph) represent the average proportion of pixels (percentage of the total pixels) at each grayscale intensity of the diaphragm ultrasound image in controls (red curve) and patients (black and grey curves). Among healthy subjects, the 95_th_ percentile for the average echodensity (grayscale value) of the whole control group was 65. Black curve represents patients with ED50 below the 50_th_ percentile and grey curve the patients with ED50 above the 50_th_ percentile (ED50 50_th_ percentile = 40) The boxplots (right graphs) represent the distribution of ED50, ED85, and HEA65 (from the left to the right) in controls and patients. Boxplot corresponds to the median with the inter-quartile range (IQR); the lower and upper whiskers extend from the hinge to the lowest and highest (respectively) values that are within 1.5 × IQR of the hinge.

Compared to healthy subjects (Fig. 1), mechanically ventilated patients exhibited higher baseline ED50 (median 27, IQR 23–34, vs 40, IQR 23–55, *p* = 0.07 for comparison) and higher baseline ED85 (median 39, IQR 36–52, vs 56, IQR 42–84, *p* = 0.04 for comparison) at the time of ICU admission. The 95th percentile of the average grayscale distribution in the healthy subjects was 65. On this basis, a grayscale value of 65 was taken as the upper limit of normal for echodensity. Eighteen patients (53%) had abnormally increased echodensity at baseline (i.e. patients with more than 5% of pixels above a grayscale value of 65 and defined as HEA65 > 5%).

In mechanically ventilated patients, baseline ED50 was not associated with patient characteristics (Table 1). Baseline ED50 was not associated with clinical outcomes such as ventilator-free days to day 60, duration of ICU admission, or death (Table 2).

**Table 2:** Clinical Outcomes in relation to baseline and change echodensity over time

Outcome	Population	Relation with baseline ED50			Relation with change in ED50 from baseline		
	*n* = 34	Coefficient	CI 95%	*p*	≤ 10-point change in ED50, * n* = 21 (62%)	> 10-point increase in ED50, * n* = 13 (38%)	*p*
Duration of ventilation (in ICU survivors), d	7 (4–11)	− 0.004	(− 0.02; 0.01)	0.55	5 (4–10)	10 (8–14)	0.10
Ventilator-free days to Day 60, d	51 (36–56)	− 0.001	(− 0.01; 0.01)	0.78	53 (49–56)	46 (0–52)	0.03
Duration of ICU admission (in ICU survivors), d	10 (5–15)	− 0.002	(− 0.02; 0.01)	0.72	10 (4–15)	11 (10–15)	0.18
Duration of hospitalization (in hospital survivors), d	26 (17–54)	0.003	(− 0.01; 0.01)	0.44	26 (16–53)	27.5 (22–72)	0.46
Maximal thickening fraction at first SBT (%), mean (SD)	29 (16)	0.001	(− 0.03; 0.03)	0.83	33 (18)	26 (13)	0.26
Complications of acute respiratory failure, *n* (%)	15 (44)	0.02	(− 0.01; 0.05)	0.27	7 (33)	8 (61)	0.21
Reintubation, *n* (%)	3 (9)	− 0.006	(− 0.07; 0.03)	0.81	1 (5)	2 (15)	0.66
Tracheostomy, *n* (%)	9 (26)	0.02	(− 0.01; 0.05)	0.28	5 (24)	4 (31)	0.96
Mechanical ventilation > 7 d, *n* (%)	18 (53)	0.02	(− 0.01; 0.05)	0.19	7 (33)	11 (85)	0.01
Readmission to ICU during same hospital admission, *n* (%)	1 (3)	− 0.04	(− 0.23; 0.04)	0.53	0 (0)	1 (8)	0.81
Death in ICU, *n* (%)	6 (18)	0.009	(− 0.02; 0.04)	0.55	2 (10)	4 (31)	0.26
Death in hospital, *n* (%)	7 (21)	0.01	(− 0.02; 0.04)	0.47	2 (10)	5 (38)	0.11

All numerical distributions are reported as median (interquartile range)

### Evolution of diaphragm echodensity

**Fig. 2:**
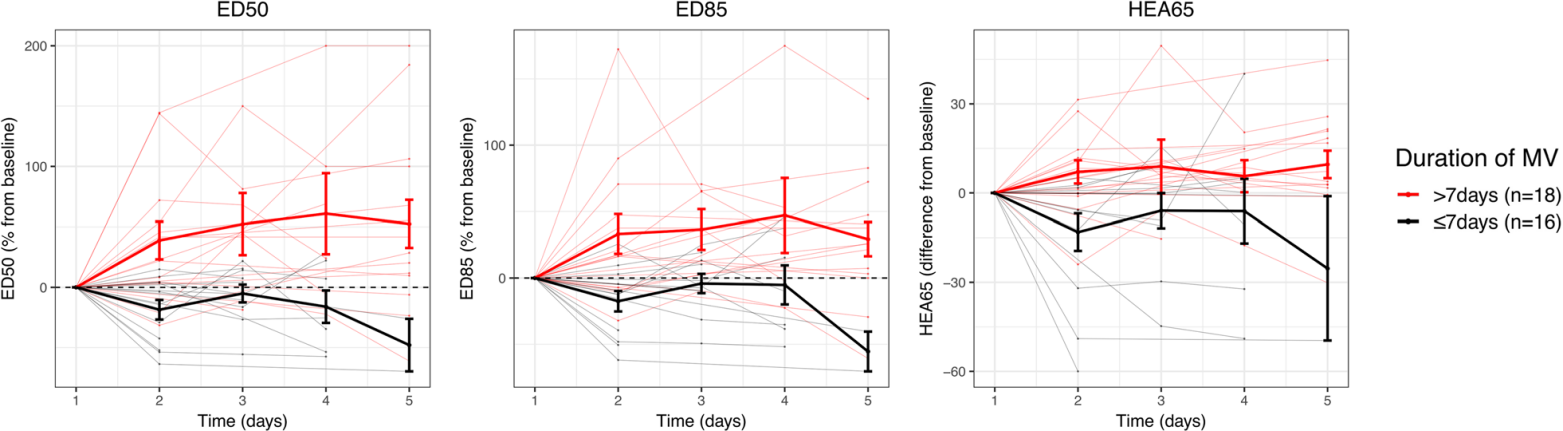
Evolution of diaphragm echodensity according to the duration of mechanical ventilation. Thin curves represent the evolution of diaphragm echodensity in each individual. Thick curves with error-bars represent mean and standard error of the mean of the diaphragm echodensity according to the groups

A median of 3 images, IQR [2–4], were available per patient. The evolution of diaphragm echodensity varied widely among patients over time during mechanical ventilation (Additional files 4 and 5, and Fig. 2). Increased diaphragm echodensity (defined a priori as > 10-point increase in ED50 from baseline) developed in 13 patients (38%) at a median of day 3 after intubation, IQR [2–4]). In these patients, the median maximal increase in ED50 reached over the first 5 days of ventilation was + 18 points (IQR + 16 to + 26 points). There was no association between patient characteristics including severity of illness score, cumulative fluid balance, or ventilator settings and the development of increased echodensity (Table 1).

**Fig. 3:**
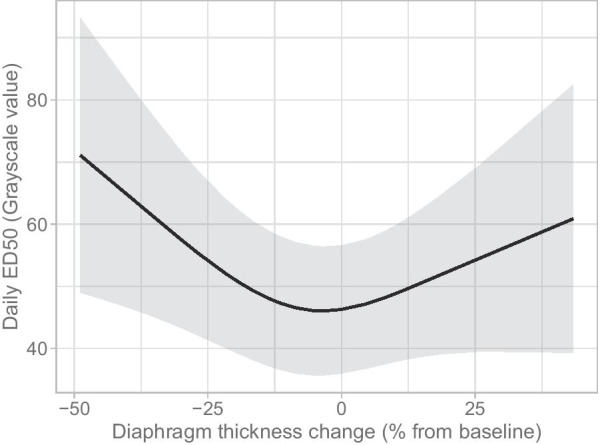
Association between change in diaphragm thickness over time and daily diaphragm echodensity value. The relationship between change in diaphragm thickness from baseline and daily ED50 value was fit using restricted cubic splines (3 knots) in linear regression and adjusted for cumulative fluid balance on day 3. The black curve represents the predicted values and grey shaded areas the 95% confident intervals

Both decreases and increases in diaphragm thickness from baseline were associated with increases in ED50 over time (*p* = 0.03, within-subjects *R*^2^ = 0.78), even after adjustment for cumulative fluid balance at day 3 (adjusted *p* = 0.03, within-subjects *R*^2^ = 0.80, Fig. 3).

In comparison to patients with unchanged echodensity, patients who developed increased echodensity had fewer ventilator-free days to day 60 (median 46, IQR 0–52, versus median 53, IQR 49–56, *p* = 0.03) and more frequently required ventilation for ≥ 7 days (85% vs. 33%, *p* = 0.01) (Table 2). There was a trend towards higher mortality in patients with increased ED50 (38% vs. 10%, *p* = 0.11). Patients who required mechanical ventilation for more than 7 days exhibited significant increases in ED50 on day 2 of mechanical ventilation (+ 40% increase from baseline, IQR + 4% to + 54%) in comparison to patients who required ventilation for less than 7 days (− 9% change in ED50 from baseline on day 2, IQR − 45% to + 4%, *p* = 0.007 for comparison) (Fig. 2). The association between increased echodensity and prolonged ventilation for ≥ 7 days persisted after adjusting for the change in diaphragm thickness from baseline (adjusted *p* = 0.01).

In a sensitivity analysis restricting to patients who remained ventilated for at least 3 days (*n* = 33) to address potential immortal time bias, increased ED50 developing within 3 days of ventilation (*n* = 8/33, 24%) was associated with fewer ventilator-free days in comparison to patients with unchanged echodensity (median 48 days, IQR [0–50] vs. median 52 days, IQR [44–56] respectively, *p* = 0.09). Among patients ventilated for at least 3 days, 88% of patients who developed increased ED50 within 3 days of ventilation (*n* = 8) required prolonged ventilation (≥ 7 days) whereas 44% of patients with no increase in ED50 (*n* = 25) required prolonged ventilation (*p* = 0.08).

## Discussion

In this preliminary description of diaphragm echodensity in mechanically ventilated patients, we found that diaphragm echodensity measurements were feasible and highly reproducible in mechanically ventilated patients. Diaphragm echodensity appears to be increased in many mechanically ventilated patients (but not all) in comparison to younger healthy subjects. Increases in diaphragm echodensity developed during the early course of mechanical ventilation in a substantial proportion of patients and these increases were associated with prolonged mechanical ventilation. Both decreases and increases in diaphragm thickness were correlated with increases in echodensity over time. Taken together, these findings suggest that sonographic measurements of echodensity represent a novel early marker of potentially important structural changes in the diaphragm associated with critical illness.

The technique for measuring echodensity of the diaphragm evaluated in this study proved highly feasible and yields results with acceptable reproducibility within and between analyzers. Echodensity was not significantly influenced by the timing of image acquisition in the respiratory cycle. One of the challenges of conducting such image analysis is to identify a single parameter that best reflects the magnitude of echodensity. Most previous studies used the mean value of the grayscale intensity as the principal marker of echodensity [3, 8, 20, 26–28]. However, the mean grayscale value may not be the most appropriate marker if the distribution of grayscale values is skewed. In our study, we employed a method similar to that used in CT-scan studies to analyze lung densitometry using the Hounsfield unit scale [29]. We assessed a number of different parameters including percentile grayscale values (50_th_ and 85_th_ percentiles, ED50 and ED85, respectively) and areas under the curve. Based on the 95_th_ percentile of an average distribution curve produced by averaging the grayscale density distributions of healthy subjects, we identified a grayscale value of 65 as the upper limit of normal. On this basis, we reported HEA65 (‘high echodensity area’ above 65), as the proportion of grayscale values above 65. These various parameters correlated quite closely. As the distribution median is the simplest and most familiar measurement, we chose to use ED50 as the primary measure of echodensity in our analysis.

Multiple previous studies have demonstrated important structural changes in the diaphragm during the early course of mechanical ventilation: decreases in diaphragm thickness suggestive of disuse atrophy [13–16] and increases in diaphragm thickness raising the hypothesis of diaphragm myotrauma from excess loading [16, 30]. These changes are associated with an impairment in diaphragm function and poor clinical outcome. To our knowledge, this is the first study to describe changes in echodensity of the diaphragm during mechanical ventilation. As we did not obtain diaphragm muscle biopsies or measure diaphragm function in these patients, the precise pathophysiological significance of these changes in echodensity—and the potential mechanistic basis for their association to prolonged ventilation—is unclear. However, radiological-pathological correlations obtained in studies of other muscles may provide insight.

Several considerations support the hypothesis that an increase in diaphragm echodensity may reflect injury to the diaphragm during critical illness. Studies in other skeletal muscles have shown a strong correlation between increased echodensity and inflammation or muscle injury confirmed with muscle biopsy [8–11]. In an experimental model of calf muscle injury in rats, the muscle degenerative phase was characterized by increased echogenicity in the injured area for up to 20 days. After the initial phase of muscle injury or myotrauma, muscle tissue may develop fibrotic changes affecting early or long-term function [9]. In neurodegenerative diseases, muscle fibrosis and dystrophy assessed by histology strongly correlated with increased echodensity [5, 26]. In mechanically ventilated patients, increases in quadriceps echodensity over time was correlated with inflammation and myonecrosis in quadriceps muscle biopsies [8]. In that study, half of the patients exhibited increased quadriceps echodensity as soon as day 1 or day 3 after ICU admission and this predicted the severity of myofiber necrosis by days 7 or 10.

Alternatively, it is possible that changes in echodensity may signify the accumulation of tissue edema [8] related to resuscitation. Changes in echodensity were unrelated to the fluid balance in this study, though edema resulting from capillary leak in the context of a systemic inflammatory response may not be perfectly captured by the fluid balance.

In the present study, an early increase of diaphragm echodensity over time during mechanical ventilation was associated with a longer duration of ventilation. It is interesting to note that changes in diaphragm echodensity appeared mostly by day 2 after ICU admission, highlighting a rapid process most likely related to the primary cause of the acute respiratory failure or possibly resulting from injurious (insufficient or excessive) respiratory effort under mechanical ventilation. Conversely, echodensity decreased from baseline in some patients over the first several days of mechanical ventilation, which could in theory suggest a rapid recovery of the initial early diaphragm injury. The possibility of injury to the lung from excess respiratory effort before intubation (referred to as patient self-inflicted lung injury [31]) has been the topic of considerable debate of late in the context of COVID-19 management. The potential for the patient to sustain an acute load-induced injury to the diaphragm before intubation is less widely appreciated. Previous studies have convincingly demonstrated that the diaphragm is vulnerable to acute load-induced injury (e.g., underassistance myotrauma from insufficient ventilator support) [32, 33] and Vassilakopoulos and colleagues have shown an acute increase in systemic inflammation derived from the diaphragm during resistive loading [34] that is relieved after initiating controlled ventilation [35]. We speculate that the increased echodensity observed at baseline in some patients in this study may potentially reflect load-induced injury sustained prior to intubation which may then progressively resolve with the institution of mechanical ventilatory support.

Increases in echodensity over time were associated with changes in diaphragm thickness, suggesting that both acute decreases and acute increases in diaphragm thickness may reflect underlying deleterious changes in muscle structure. We observed increased echodensity at admission (compared to healthy subjects) and early changes during the ICU course. Thus, early change in diaphragm echodensity could be an interesting early marker of diaphragm injury to be studied as part of future diaphragm-protective ventilation trials [36]. The relation between changes in echodensity and outcome was independent of the change in thickness (itself associated with prolonged ventilation [16]), suggesting that this measure may have added prognostic value compared to diaphragm thickness at a given time.

This study has several important limitations. Adjustable settings such as gain or depth can have an effect on the grayscale value of the image produced [27] and ideally should be standardized for all images taken in all patients. The angle at which the probe is held, the pressure with which the probe is held against the body, as well as the amount of subcutaneous adipose tissue or edema between the probe and the muscle are all recognized as potential factors that affect the image processing (and thus the grayscale value of the image produced) [3, 37–39]. To minimize measurement noise within patients related to the ultrasound settings, we selected images with consistent gain and depth in each patient and analyzed echodensity changes over time expressed relative to the baseline value in each patient. However, it was impossible to obtain the exact same settings between patients, limiting the validity of comparison between patients.

Reproducibility was assessed within and between analyzers from a single diaphragm image. Inter-observer reproducibility for ED measurement from different ultrasound images remains to be determined in future studies. Because this was a secondary analysis of a convenience sample from a previous cohort study, a sample size calculation was not performed. We included all patients for whom we possessed stored images of high quality with consistent settings (i.e. unchanged gain or frequency settings between different study days). The results of this are hypothesis-generating and require confirmation in future prospective studies designed to further correlate changes in diaphragm echodensity to markers of systemic inflammation (cytokines, etc.), diaphragm histology, diaphragm muscle function, and clinical outcomes.

The systematic difference in echodensity between mechanically ventilated patients and healthy subjects may arise from differences in age or chronic comorbidity between groups, apart from any acute muscle injury in mechanically ventilated patients. Both comorbidity and age have been shown to affect muscle echodensity [8, 28, 40]. These factors might account for some of the observed differences between patients and healthy subjects in this study; to mitigate confounding in this comparison, we analyzed the change in echodensity within subjects over time and normalized patients to their baseline echodensity.

## Conclusion

Diaphragm echodensity can be measured feasibly and reproducibly in mechanically ventilated patients. In this study, diaphragm echodensity was increased in mechanically ventilated patients compared to healthy subjects and increases in echodensity over time were associated with prolonged mechanical ventilation. Both decreases and increases in diaphragm thickness over time were associated with increases in echodensity over time. Diaphragm echodensity merits further investigation as a potential clinically relevant marker of muscle injury during critical illness.

## Supplementary Information

**Additional file 1** Method of analysis of the diaphragm echodensity.** A**. Panel of grayscale, from black (grayscale value = 0) to white (grayscale value = 255). **B**. Example of a diaphragm ultrasound image (Dia: diaphragm). The yellow rectangle delineates the diaphragm area (excluding the pleural and peritoneal membranes). C. Example of a histogram in a control healthy subject representing the proportion of pixels (percentage of the total pixels) at each grayscale intensity of the diaphragm. The two right straight lines represent the grayscale intensity at 50th (ED50, blue line) and 85th (ED85, red line) percentile of the total pixels. The green colored area represents the proportion of pixels (percentage of the total pixels) above the grayscale intensity of 65 (green area) and considered in our study as the upper limit of normal for echodensity. ED: echodensity; HEA: high echodensity area.

**Additional file 2** Bland-Altman plot of repeated measurements of diaphragm echodensity. The analyses were performed with the median grayscale value of the histogram (i.e grayscale intensity at 50th percentile of the total pixels = ED50). The blue line indicates bias, the dashed lines indicate both limits of agreement. The x-axis shows the mean of two values. The y-axis shows the difference between means of these values. The blue lines represent the bias, the dashed lines indicate both limits of agreement.** A**: Between-analyzer reproducibility of echogenicity (measurement on one image, two analyzers): bias = -1.5, limits (-8.6; 5.7); n=30 images.** B**: Between-image reproducibility of echogenicity (measurement on two separate images collected on the same patient on the same day, single analyzer): bias = -2.8, limits (-15.8; 10.2); n=30 images.** C**: Reproducibility of echodensity at end-expiration and end-inspiration (2 measurements on the same respiratory cycle, single analyzer): bias = -1.3, limits (-9.8; 7.2); n=15 images.

**Additional file 3** Bland-Altman plot of repeated measurements of diaphragm echodensity according to the ultrasound machine (Philips, Mindray, Sonosite). The analyses were performed with the median grayscale value of the histogram (i.e grayscale intensity at 50th percentile of the total pixels = ED50).

**Additional file 4** Examples of two patients with and without a change in diaphragm echodensity over time.** A**. Patient without any specific change in diaphragm echodensity over time who received 3 days of mechanical ventilation.** B**, Patient with changes in diaphragm echodensity over time who received 10 days of mechanical ventilation.

**Additional file 5** Evolution of diaphragm echodensity according to the duration of mechanical ventilation. Thin curves represent the evolution of diaphragm echodensity in each individual. Thick curves with error-bars represent mean and standard error of the mean of the diaphragm echodensity according to the groups.

## Data Availability

The datasets used and/or analysed during the current study are available from the corresponding author on reasonable request.

## Abbreviations

ED50: echodensity at the 50th percentile
ED85: echodensity at the 85_th_ percentile
HEA65: high echodensity area above grayscale value of 65
ICU: intensive care unit
IQR: interquartile ranges
SAPS II: simplified acute physiology score II
SAS: riker sedation-agitation scale
SOFA: sequential organ failure assessment
