# Supplementary figures and images for: Diaphragm echodensity in mechanically ventilated patients: a description of technique and outcomes
Source: Crit Care. 2021 Feb 16;25:64. doi: 10.1186/s13054-021-03494-9 (PMC7884870; doi:10.1186/s13054-021-03494-9)

**A**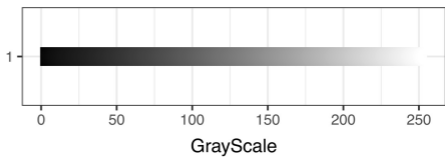**B**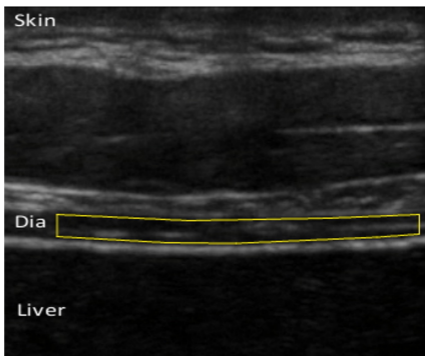**C**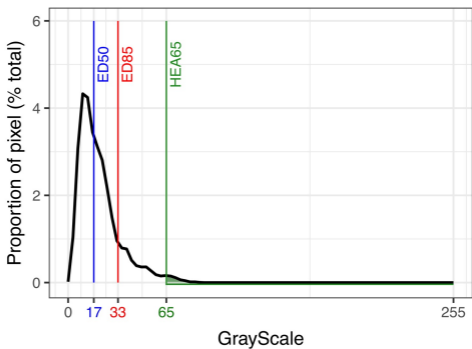

Supplement: Supplementary file 1 — Additional file 1 Method of analysis of the diaphragm echodensity. A. Panel of grayscale, from black (grayscale value = 0) to white (grayscale value = 255). B. Example of a diaphragm ultrasound image (Dia: diaphragm). The yellow rectangle delineates the diaphragm area (excluding the pleural and peritoneal membranes). C. Example of a histogram in a control healthy subject representing the proportion of pixels (percentage of the total pixels) at each grayscale intensity of the diaphragm. The two right straight lines represent the grayscale intensity at 50th (ED50, blue line) and 85th (ED85, red line) percentile of the total pixels. The green colored area represents the proportion of pixels (percentage of the total pixels) above the grayscale intensity of 65 (green area) and considered in our study as the upper limit of normal for echodensity. ED: echodensity; HEA: high echodensity area. [file 13054_2021_3494_MOESM1_ESM.pdf]

**A**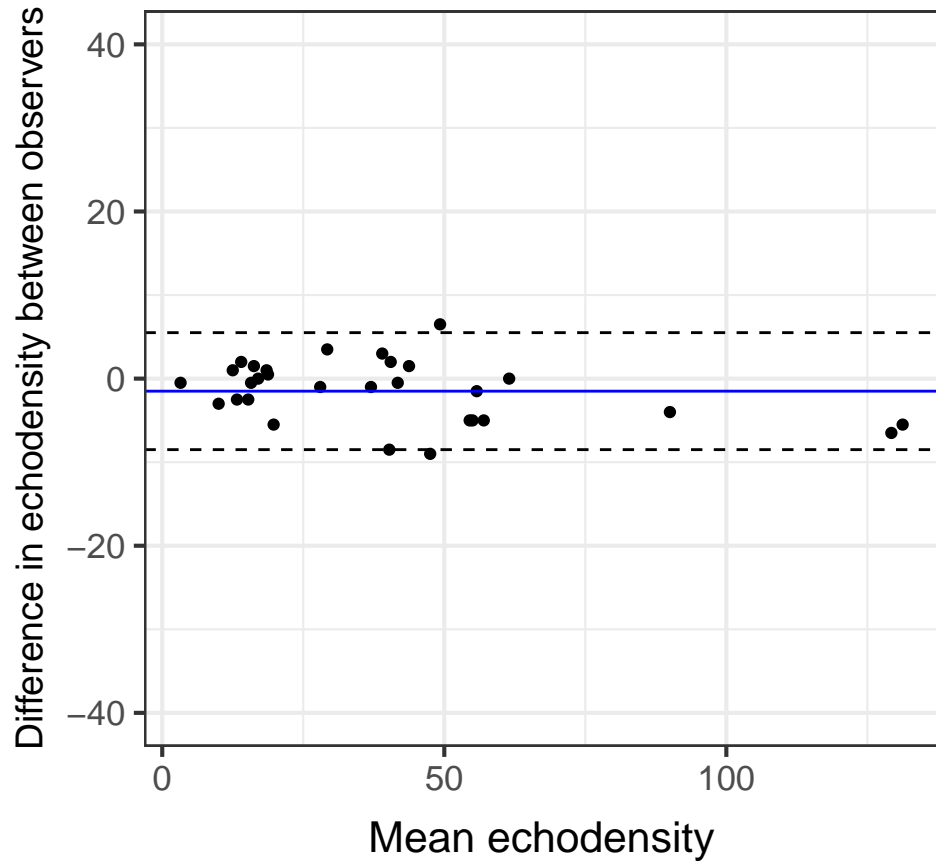**B**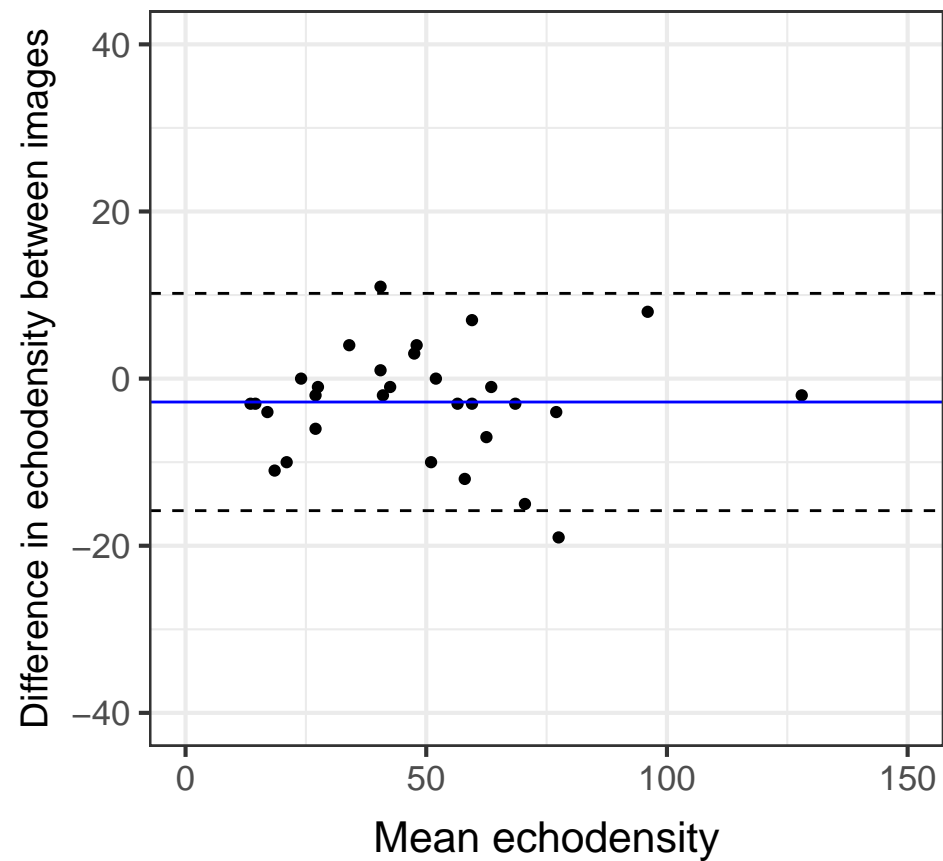**C**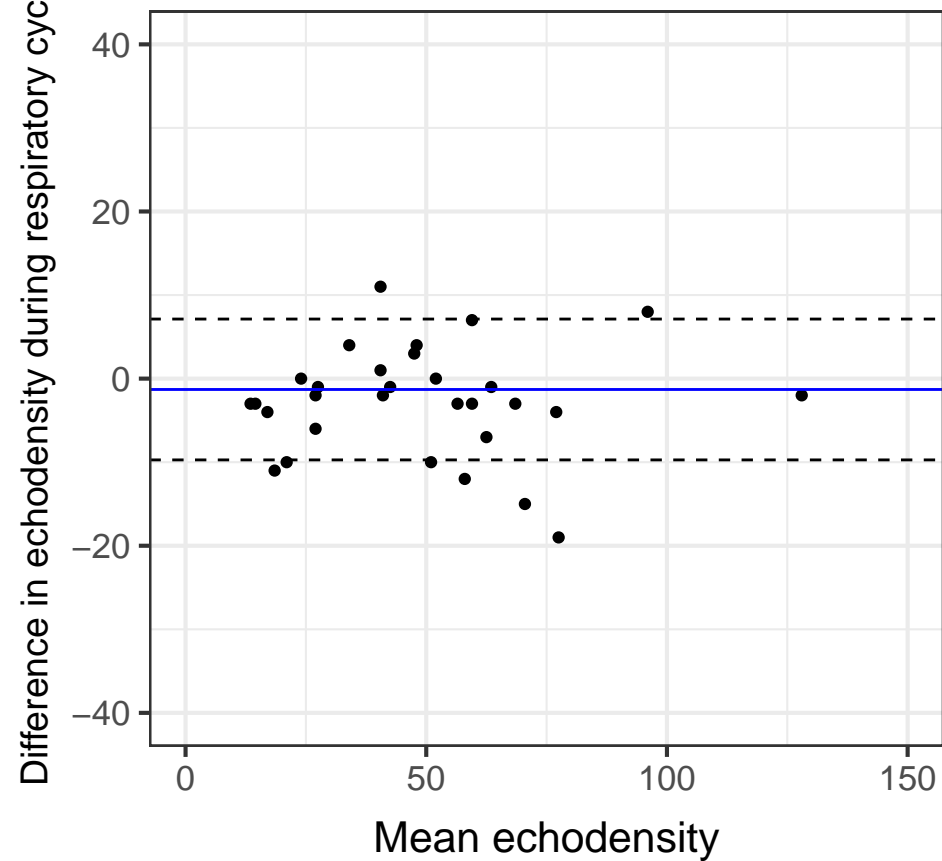

Supplement: Supplementary file 2 — Additional file 2 Bland-Altman plot of repeated measurements of diaphragm echodensity. The analyses were performed with the median grayscale value of the histogram (i.e grayscale intensity at 50th percentile of the total pixels = ED50). The blue line indicates bias, the dashed lines indicate both limits of agreement. The x-axis shows the mean of two values. The y-axis shows the difference between means of these values. The blue lines represent the bias, the dashed lines indicate both limits of agreement. A: Between-analyzer reproducibility of echogenicity (measurement on one image, two analyzers): bias = -1.5, limits (-8.6; 5.7); n=30 images. B: Between-image reproducibility of echogenicity (measurement on two separate images collected on the same patient on the same day, single analyzer): bias = -2.8, limits (-15.8; 10.2); n=30 images. C: Reproducibility of echodensity at end-expiration and end-inspiration (2 measurements on the same respiratory cycle, single analyzer): bias = -1.3, limits (-9.8; 7.2); n=15 images. [file 13054_2021_3494_MOESM2_ESM.pdf]

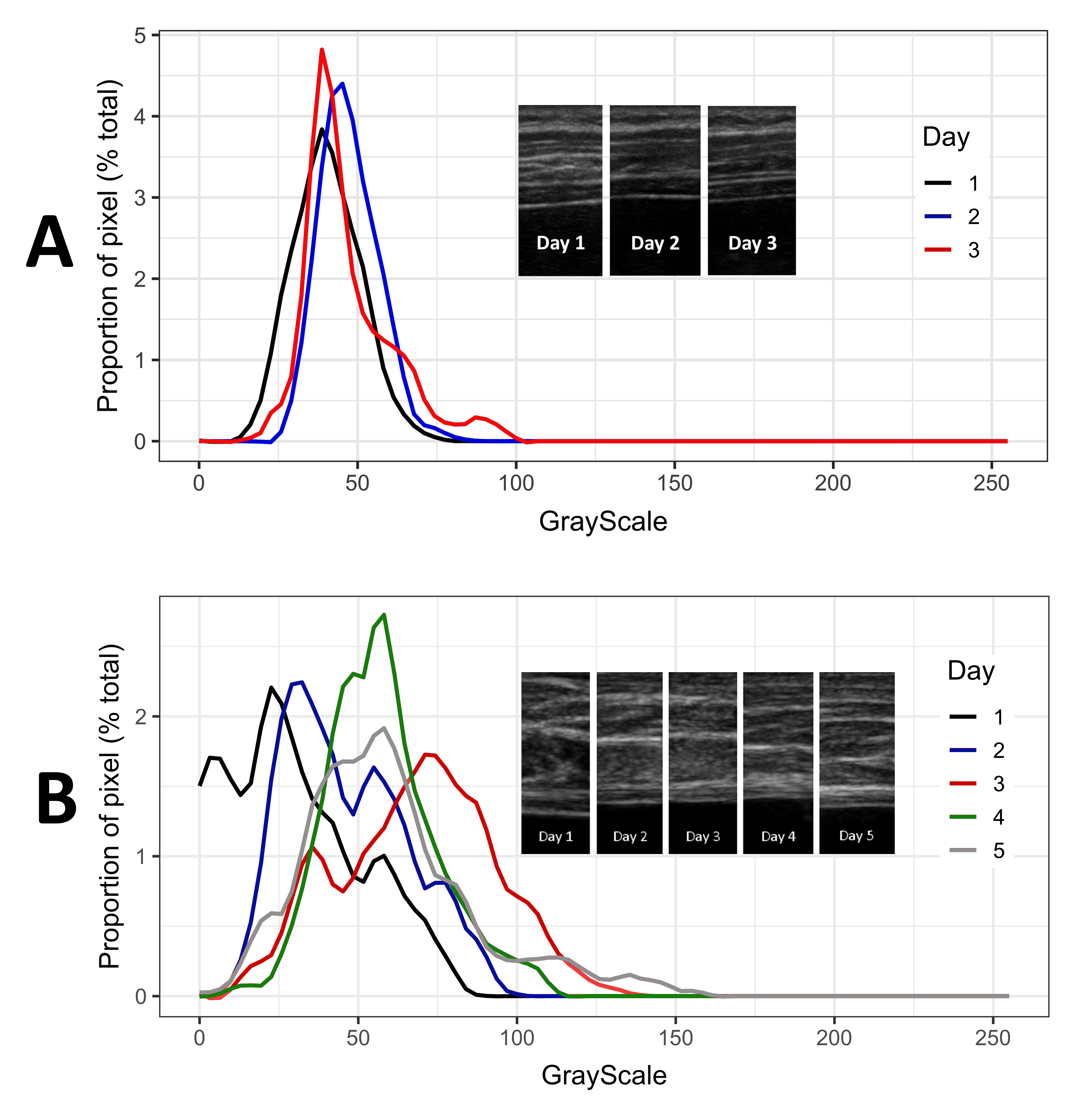

Supplement: Supplementary file 4 — Additional file 4 Examples of two patients with and without a change in diaphragm echodensity over time. A. Patient without any specific change in diaphragm echodensity over time who received 3 days of mechanical ventilation. B, Patient with changes in diaphragm echodensity over time who received 10 days of mechanical ventilation. [file 13054_2021_3494_MOESM4_ESM.tiff]

ED50

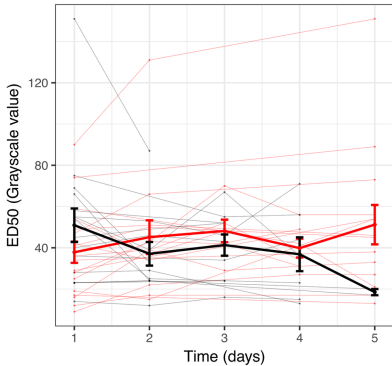

ED85

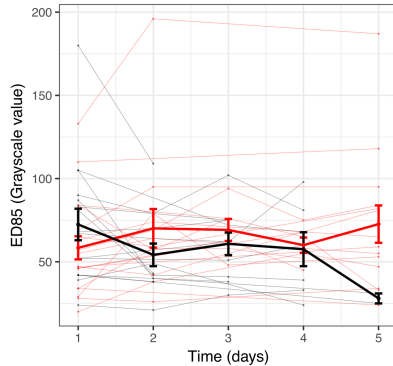

HEA65

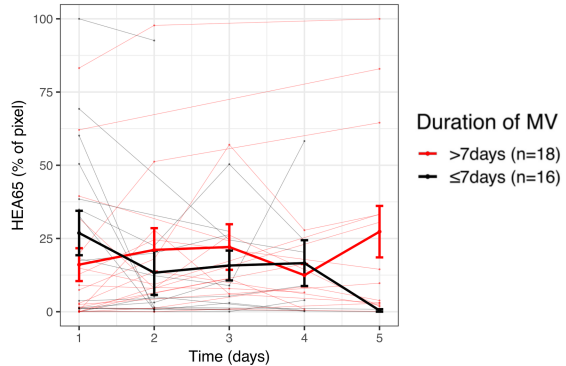

Supplement: Supplementary file 5 — Additional file 5 Evolution of diaphragm echodensity according to the duration of mechanical ventilation. Thin curves represent the evolution of diaphragm echodensity in each individual. Thick curves with error-bars represent mean and standard error of the mean of the diaphragm echodensity according to the groups. [file 13054_2021_3494_MOESM5_ESM.pdf]
